# Clinical benefit and safety of immunotherapy beyond progression in advanced and locally advanced lung cancer: a real-world cohort study

**DOI:** 10.3389/fonc.2026.1796804

**Published:** 2026-04-28

**Authors:** Rui Huangfu, Hua Lan, Rongjie Li, Kun Hou, Yaodong Ping

**Affiliations:** 1 School of Pharmacy, Inner Mongolia Medical University, Hohhot, China; 2 Department of Pharmacy, Peking University Cancer Hospital Inner Mongolia Hospital, Hohhot, China; 3 Department of Pharmacy, Key laboratory of Carcinogenesis and Translational Research (Ministry of Education/Beijing), Peking University Cancer Hospital & Institute, Beijing, China

**Keywords:** disease progression, advanced or locally advanced lung cancer, immune checkpoint inhibitor, immune treatment beyond progression, safety and efficacy

## Abstract

**Objective:**

To evaluate the efficacy and safety of immune treatment beyond progression (ITBP) strategies in patients with advanced or locally advanced lung cancer.

**Methods:**

A retrospective analysis was conducted on clinical data from 166 lung cancer patients who received ITBP, defined as continuation of the immune checkpoint inhibitor (ICIs) beyond disease progression, at Inner Mongolia Hospital of Peking University Cancer Hospital between January 2023 and December 2025. Data collected included patient clinical characteristics, number of prior treatment lines, treatment regimens, and best overall response. The primary endpoint was progression-free survival (PFS) and Overall Survival (OS). Secondary endpoints included disease control rate (DCR), objective response rate (ORR), and the incidence of immune-related adverse events (irAEs).

**Results:**

A total of 166 patients were enrolled in the study, including 82 in the ITBP group and 84 in the NTBP group. The ORR was significantly higher in the ITBP group compared to the NTBP group (17.1% vs. 0.60%, p = 0.024), whereas no statistically significant difference in DCR was observed between the two groups (86.6% vs. 76.2%, p = 0.086). ITBP treatment was associated with a significant prolongation of OS (HR = 0.48, 95% CI: 0.23–1.00, p = 0.046) and PFS (HR = 0.69, 95% CI: 0.49–0.8, p = 0.027). Subgroup analyses revealed that patients aged ≥60 years, those with stage IV disease, non-squamous histology, absence of liver metastasis, and those who achieved a PFS of ≥6 months after prior-line therapy derived greater clinical benefit from ITBP. Both univariate and multivariate analyses consistently demonstrated that ITBP, as a therapeutic strategy, was independently associated with reduced risk of disease progression, with statistical significance. Safety analyses indicated that the incidence of most adverse events was comparable between groups, with no significant differences noted.

**Conclusion:**

Compared with NTBP, ITBP significantly improves ORR and prolongs OS and PFS in lung cancer patients, with particularly pronounced benefits observed in predefined subgroups. Furthermore, ITBP exhibits a favorable safety profile without increasing the risk of severe adverse events.

## 1 Introduction

Primary bronchogenic lung cancer, commonly referred to as lung cancer, is one of the most prevalent and deadly cancers worldwide, including in China (1). In 2022, lung cancer was the leading cause of new cases and deaths from malignant tumors in China (2). Lung cancer is divided into small-cell (SCLC) and non–small-cell (NSCLC) lung cancer, with NSCLC accounting for more than 85% of cases. Early-stage lung cancer typically presents with no obvious symptoms, and most patients are diagnosed at advanced stages, resulting in a 5-year survival rate of approximately 20% for those with advanced disease (3). Therefore, identifying effective treatment strategies is critical.

Immunotherapy has been shown to alter the biological characteristics of tumors, allowing for sustained survival benefits even after imaging progression, a phenomenon referred to as post-progression prolongation of survival (PPPS). Although some patients exhibit disease progression in imaging, clinical symptoms may improve, likely due to enduring antitumor immune responses, including continuous recognition and memory of tumor antigens. As cycles of antitumor immunity repeat, the immune response may strengthen, offering potential long-term benefits (4). This suggests that continuing immune treatment beyond progression (ITBP) could be a promising strategy in cancer treatment.

In oncology, ITBP refers to continuing immunotherapy, with either the same or different immune checkpoint inhibitors (ICIs), after disease progression during previous ICIs treatment. This approach aims to maintain immunotherapy despite tumor progression, tailored to each patient’s condition. In cases of slow progression or pseudo-progression, continuing ITBP could be beneficial, whereas it is not recommended for patients whose condition deteriorates after progression. Secondary resistance could be addressed by combining ITBP with oncolytic viruses or anti-angiogenic therapies, potentially improving immune resistance and reactivating immune function (5).

Initial studies such as KEYNOTE 010 demonstrated that patients who experienced disease progression after 35 cycles of pembrolizumab treatment still benefited from further pembrolizumab therapy, with an ORR of 35% and a DCR of 85%. Furthermore, these patients achieved a 5-year OS rate of approximately 85% (6). In a recent Phase III study conducted by Professor Gandara’s team in the United States (the OAK study), patients who continued atezolizumab after progression had a median OS of 12.7 months, compared with 8.8 months in those who switched to docetaxel (NTBP). This suggests that continued treatment with atezolizumab post-progression is associated with better outcomes and superior tolerability than docetaxel (7). Some scholars conducted a retrospective analysis of treatment outcomes in patients with lung cancer who received ITBP at Peking Union Medical College Hospital between 2018 and 2019. The results demonstrated that OS was significantly prolonged among patients who continued immunotherapy beyond PD. Furthermore, the study revealed that clinical symptom improvement occurred even in patients whose tumor mutational burden (TMB) did not decrease (8). However, contrasting results were reported in a cohort study from Japan, which included 15 patients with NSCLC who received PD-1 inhibitors, as crossover therapy after first-line PD-L1 inhibitor treatment did not demonstrate significant survival benefits. The divergent findings between these two studies may be attributed to differences in patient characteristics, particularly because the Japanese study included only patients with low PD-L1 expression (PD-L1 < 50%), which may have contributed to the suboptimal efficacy of ITBP (9). A real-world study conducted by Stinchcombe (10) involving 4,223 patients further substantiated the efficacy of ITBP, showing better survival outcomes in patients receiving immunotherapy beyond progression (OS: ITBP vs NTBP, 11.5 vs 5.1 months, HR 0.69, P < 0.001).

Real-world studies (RWS) can more comprehensively reflect therapeutic efficacy and safety by collecting patient data from routine clinical practice, thereby addressing the limitations imposed by the strict inclusion and exclusion criteria of randomized controlled trials. In this context, this retrospective study utilized real-world data to evaluate the clinical efficacy and safety of ITBP in patients with locally advanced or advanced lung cancer, providing robust evidence to support clinical decision-making and facilitate the optimization of individualized treatment strategies.

## 2 Materials and methods

### 2.1 Study design and patients

A total of 166 lung cancer patients who had received first-line ICIs and subsequently experienced PD were retrospectively enrolled from the Inner Mongolia Hospital of Peking University Cancer Hospital between January 1, 2023, and June 30, 2025. After progression, patients who continued ITBP—defined as receiving the same or a different ICIs regimen, either as monotherapy or in combination with other treatments—were included in the ITBP group. In contrast, patients who discontinued ICIs and switched to conventional therapies, such as chemotherapy or targeted therapy, were assigned to the non-immunotherapy beyond progression (NTBP) group. Clinical data, imaging characteristics, and relevant laboratory test results were collected for both groups. ICIs included cytotoxic T-lymphocyte-associated antigen 4 (CTLA-4), programmed cell death protein 1 (PD-1), and programmed death-ligand 1 (PD-L1) inhibitors.

### 2.2 Criteria for inclusion and exclusion

The inclusion criteria were as follows: (I) patients with NSCLC at TNM stage IIIA–IV according to the eighth edition of the American Joint Committee on Cancer (AJCC) tumor–node–metastasis classification, or those with extensive-stage SCLC, as well as other histologically confirmed types of locally advanced or advanced lung cancer; (II) PD after first-line ICIs therapy, assessed according to Response Evaluation Criteria in Solid Tumours version 1.1 (RECIST 1.1), followed by continuation of ICIs treatment; (III) presence of measurable lesions; (IV) Eastern Cooperative Oncology Group (ECOG) performance status score of ≤2; and (V) availability of complete clinical data, including clinical characteristics, imaging examinations, and laboratory test results.

Exclusion criteria were: (I) presence of concurrent malignancies at other sites; (II) pre-existing immune deficiencies unrelated to cancer, or long-term use of glucocorticoids or immunosuppressive agents; (III) pregnant or lactating women; (IV) ECOG performance status score >2; and (V) incomplete medical records or missing imaging data.

### 2.3 Data collection

The RWS data were collected from Peking University Cancer Hospital and Inner Mongolia Hospital of Peking University Cancer Hospital. Research data were extracted from the hospital’s HIS (Hospital Information System) to obtain baseline clinicopathological characteristics, including patient registration information (name, age, gender, height, weight), smoking and alcohol consumption history, pathological subtypes, immunotherapy agents used, and number of treatment cycles. Safety data primarily comprised irAEs. In this study, irAEs information was mainly derived from clinical documentation in the electronic medical record system, such as physician progress notes, discharge summaries, ancillary test reports, and pharmaceutical care records. Due to the fact that some adverse events in real-world settings lacked clear grading according to the Common Terminology Criteria for Adverse Events (CTCAE), this study did not perform strict classification of irAEs severity. Instead, only the occurrence and types of irAEs were recorded, to comprehensively reflect the overall safety profile of immune checkpoint inhibitor cross-line therapy in routine clinical practice.

The primary endpoints were OS and PFS.To ensure a consistent time origin and minimize potential bias, documented PD was used as the starting point for survival analyses in both groups.OS was defined as the time from PD to death from any cause or the last follow-up. PFS was defined as the time from PD to further disease progression after post-progression treatment or death, whichever occurred first. The secondary observation indicators included ORR and DCR. ORR is calculated as the sum of complete response CR and PR, while DCR encompasses CR, PR, and SD. The last follow-up date was June 30, 2025.

### 2.4 Ethics statement

This study was approved by the Ethics Committee of the Inner Mongolia Hospital of Peking University Cancer Hospital (Approval No. WZ202527). Due to the retrospective nature of the study, the requirement for informed consent was waived.

### 2.5 Statistical analysis

All data analyses in this study were performed using SPSS 27.0 and R 4.4.2 software. The statistical significance level was set at two-sided α = 0.05, with p < 0.05 considered statistically significant. Patients were classified into the ITBP and NTBP groups according to their treatment modality. Baseline characteristics between the two groups were compared using the chi-square test or Fisher’s exact test for categorical variables and the Student’s t-test or Mann–Whitney U test for continuous variables, as appropriate. In addition, standardized mean differences (SMDs) were calculated to assess the balance of baseline covariates, with an SMD < 0.1 indicating negligible imbalance and < 0.2 considered acceptable. Kaplan-Meier survival curves were generated using the “survival” and “survminer” packages in R, and group differences in overall survival OS and PFS were assessed using the Log-rank test. The Cox proportional hazards regression model was employed to evaluate the association between clinical variables and PFS. A two-step modeling approach was adopted: first, univariate analysis was conducted to identify candidate variables associated with PFS (p < 0.10), which were subsequently included in the multivariate Cox regression model to determine independent predictors of PFS. All clinical variables were treated as categorical variables. Statistical significance was defined as p < 0.05.

## 3 Results

### 3.1 Patient clinical characteristics

**Figure 1:**
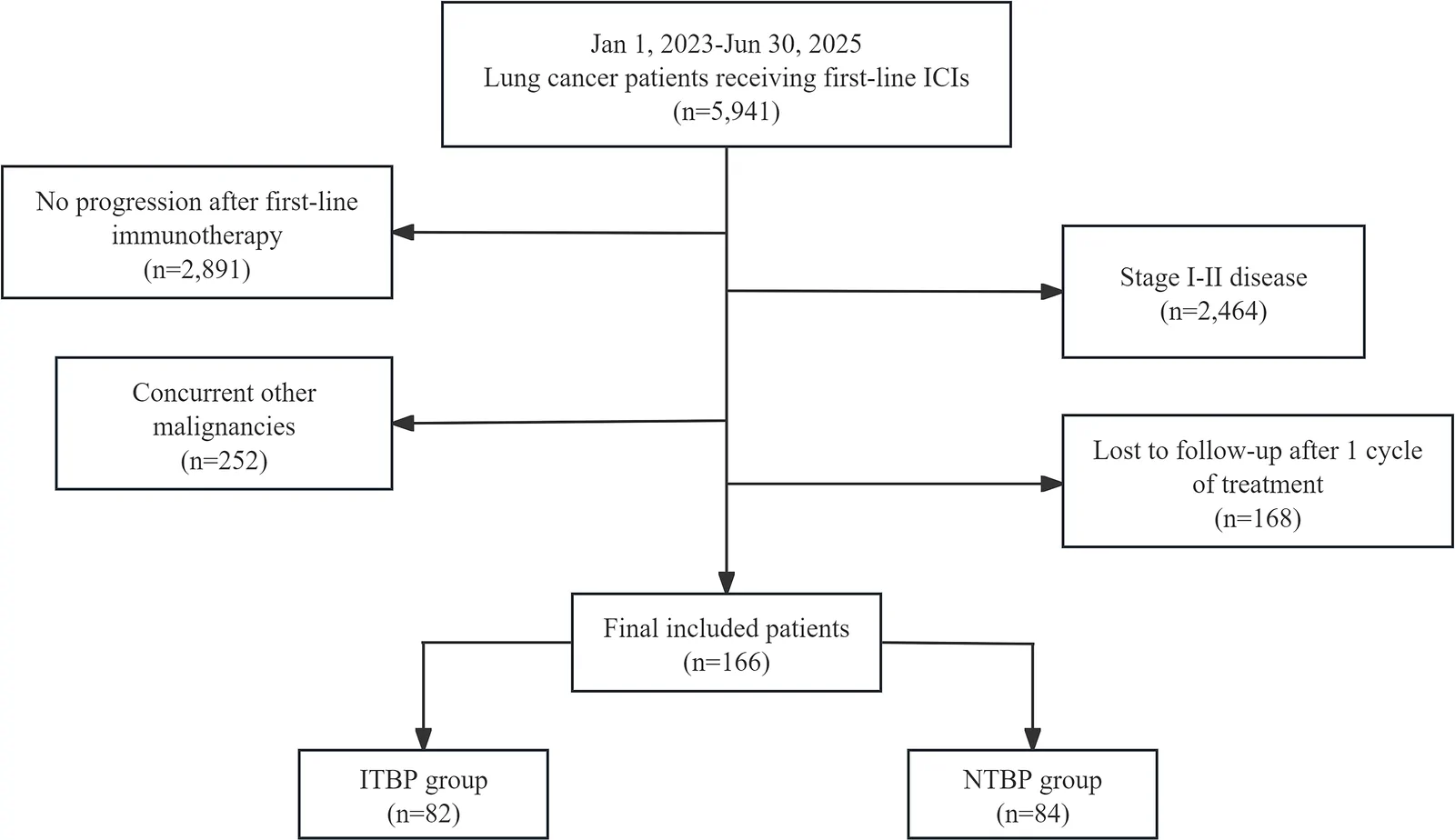
Flowchart of this study.

This study utilized the electronic medical record system of the Inner Mongolia Hospital of Peking University Cancer Hospital to identify lung cancer patients treated at our institution between January 1, 2023, and June 30, 2025. A preliminary screening identified a total of 5,941 lung cancer patients who were treated with first-line ICIs. Following predefined inclusion and exclusion criteria, we ultimately included 166 eligible patients in our analysis. Based on post-progression treatment strategies, patients were categorized into two groups: ITBP group and the NTBP group. The ITBP group included 82 patients who continued ICIs after PD during first-line treatment, while the remaining 84 patients who discontinued ICIs and switched to alternative treatments were assigned to the NTBP group. The patient selection process is illustrated in **Figure 1**.

**Table 1** summarizes the baseline characteristics of the two patient groups. Overall, patients aged ≥60 years accounted for 72.3% of the cohort. A total of 143 patients (86.1%) were male and 23 (13.9%) were female. Most patients had a history of smoking (122 patients, 73.5%). Regarding disease stage, 34 patients (20.5%) had locally advanced disease, while 132 patients (79.5%) had advanced disease. In terms of histological subtype, 66 patients (39.8%) had squamous cell carcinoma, 59 patients (35.5%) had NSCLC, and 41 patients (24.7%) had SCLC. With respect to molecular characteristics, 37 patients (22.3%) were positive for driver gene mutations, and 30 patients (18.1%) exhibited high PD-L1 expression (≥50%). Approximately 30% of the patients presented with distant metastases. Regarding the best response to first-line immunotherapy, 93 patients (56.0%) achieved PR, 71 patients (42.8%) achieved SD, and only 2 patients (1.2%) experienced PD. In terms of treatment regimens, PD-1 inhibitors were used as first-line therapy in 134 patients (80.3%), whereas PD-L1 inhibitors were administered in 32 patients (19.7%). For first-line PFS, 50 patients (30.1%) had PFS < 6 months, while 116 patients (69.9%) had PFS ≥ 6 months. Comparisons of baseline characteristics between the two groups were performed using both p values and standardized mean differences (SMDs). Overall, most variables were well balanced between the two groups (SMD < 0.2), although some degree of imbalance was observed in certain variables, particularly liver metastasis (SMD = 0.340).

**Table 1:** Baseline characteristics of the patients.

Baseline characteristics	ITBP N = 82(%)	NTBP N = 84(%)	*p*	SMD
Age			0.345	0.148
≥60	62 (75.6)	58 (69.0)		
<60	20 (24.4)	26 (31.0)		
Gender			0.131	0.236
Male	74 (90.2)	69 (82.1)		
Female	8 (9.8)	15 (17.9)		
Smoking history			0.656	0.068
Yes	59 (72.0)	63 (75.0)		
No	23 (28.0)	21 (25.0)		
Stage			0.643	0.074
IIIA/B/C	18 (22.0)	16 (19.0)		
IVA/B	64 (78.0)	68 (81.0)		
Histology			0.087	0.238
NSCLC	57(69.5)	67 (79.8)		
SCLC	25 (30.5)	17 (20.2)		
Bone metastasis			0.394	0.160
Yes	26 (31.7)	33 (39.3)		
No	56 (68.3)	51 (60.7)		
Brain metastasis			0.572	0.044
Yes	29 (35.4)	28 (33.3)		
No	53 (64.6)	56 (66.7)		
Liver metastasis			0.056	0.340
Yes	30 (36.6)	18 (21.4)		
No	52 (63.4)	66 (78.6)		
Best response to previous line			0.114	0.248
PR	51 (62.2)	42 (50.0)		
SD/PD	31 (37.8)	42 (50.0)		
PFS of first-line			0.361	0.142
PFS<6m	22 (26.8)	28 (33.3)		
PFS≥6m	60 (73.2)	56 (66.7)		

### 3.2 Efficacy evaluation

#### 3.2.1 ORR and DCR

Short-term efficacy of ITBP was evaluated in both groups. No patients achieved complete response (CR) in either group. In the ITBP group, partial response (PR) was observed in 14 patients (17.1%), stable disease (SD) in 57 patients (69.5%), and progressive disease (PD) in 11 patients (12.6%). In the NTBP group, PR occurred in 5 patients (6.0%), SD in 59 patients (70.2%), and PD in 20 patients (23.8%). The objective response rate (ORR) was significantly higher in the ITBP group compared with the NTBP group (17.1% vs. 6.0%, p = 0.024), whereas no significant difference was observed in disease control rate (DCR) between the groups (86.6% vs. 76.2%, p = 0.086) (**Table 2**).

**Table 2:** The tumor response of all patients in the second-line treatment.

Response	ITBP group (N=82)	NTBP group (N=84)	*p*
CR, n (%)	0 (0)	0 (0)	
PR, n (%)	14 (17.1)	5 (6.00)	
SD, n (%)	57 (69.5)	59 (70.2)	
PD, n (%)	11 (13.4)	20 (23.8)	
ORR, n (%)	14 (17.1)	5 (6.00)	0.024
DCR, n (%)	71 (86.6)	64 (76.2)	0.086

CR, complete response; PR, partial response; SD, stable disease; PD, progressive disease; ORR, objective response rate; DCR, disease control rate; ITBP, immunotherapy beyond progression.

#### 3.2.2 OS and PFS

**Figure 2:**
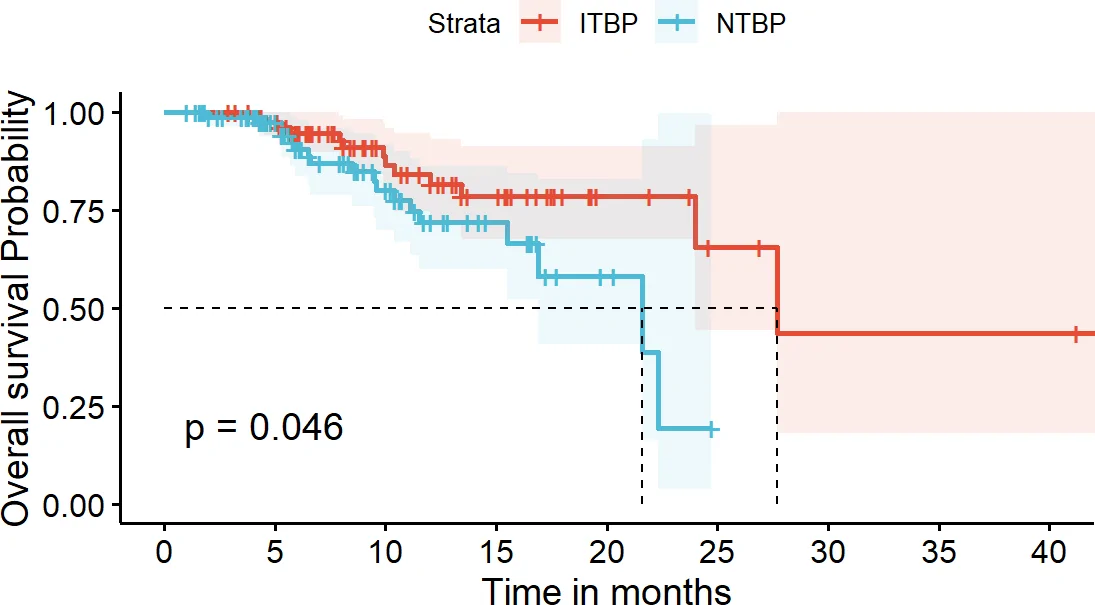
Kaplan-meier survival curves of OS in the ITBP group and the NTBP group.

A total of 82 patients in the ITBP group and 84 patients in the NTBP group were included in the analysis. Kaplan–Meier survival analysis demonstrated that the median OS in the ITBP group was 27.7 months (95% CI: 24.0–NA), which was significantly longer than that in the NTBP group (21.6 months; 95% CI: 16.9–NA). Cox proportional hazards regression analysis indicated that patients in the ITBP group had an approximately 52% lower risk of death compared with those in the NTBP group (HR = 0.48, 95% CI: 0.23–1.00, p = 0.046) (**Figure 2**), and the difference was statistically significant.

**Figure 3:**
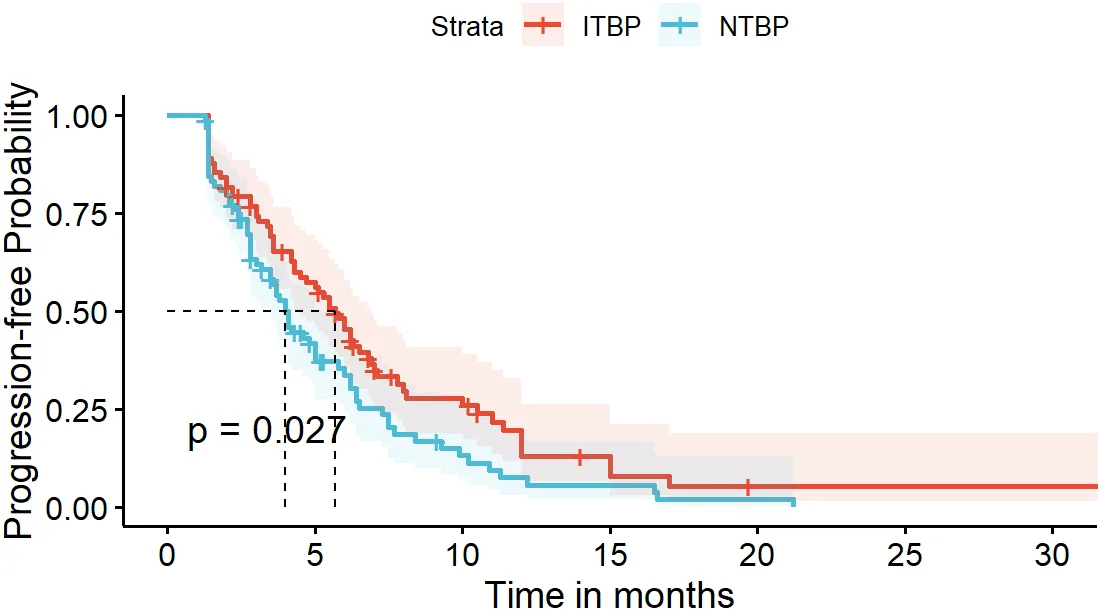
Kaplan-meier survival curves of PFS in the ITBP group and the NTBP group.

With respect to PFS, the median PFS was 5.7 months (95% CI: 4.5–6.9) in the ITBP group and 4.0 months (95% CI: 3.5–5.0) in the NTBP group. The ITBP regimen was associated with a significantly reduced risk of disease progression (HR = 0.69, 95% CI: 0.49–0.86, p = 0.027) (**Figure 3**).

Overall, these findings indicate that, compared with the NTBP regimen, ITBP treatment confers significant advantages in prolonging OS and improving PFS, suggesting potential long-term survival benefits and meaningful clinical value for this patient population.

#### 3.2.3 Treatment effects by lung cancer subtype

Although the present study demonstrated that ITBP was associated with survival benefits in the overall lung cancer population, substantial differences exist between SCLC and NSCLC with respect to biological behavior, patterns of PD, and treatment sensitivity. Accordingly, ITBP strategies for these two disease entities differ considerably. SCLC is characterized by high aggressiveness, rapid PD, and an initial sensitivity to systemic therapy, followed by a high propensity for early relapse. As a result, ITBP in SCLC primarily focuses on modifications of chemotherapy regimens, with the exploratory incorporation of immunotherapy in selected patients. In contrast, NSCLC exhibits marked molecular heterogeneity, and ITBP strategies are more frequently centered on the optimization and sequencing of targeted therapy, immunotherapy, and combination regimens.

Therefore, it is essential to further investigate the differential efficacy and shared or distinct therapeutic strategies of ITBP between patients with SCLC and NSCLC from a histological perspective. Such analyses may provide a more robust evidence base to support individualized treatment decision-making and improve clinical outcomes.

**Figure 4:**
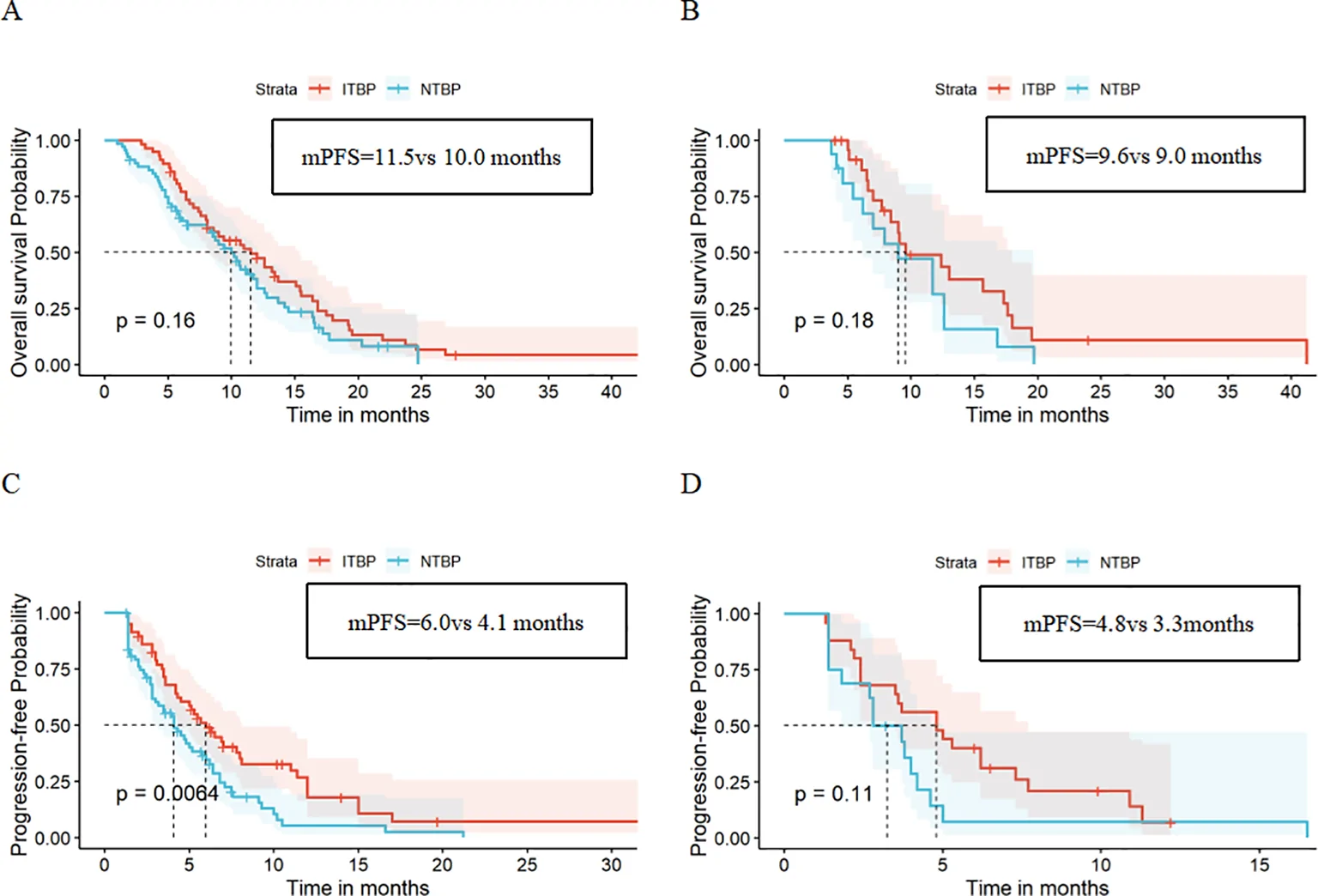
K-M Analysis of OS and PFS by pathological type (ITBP vs. NTBP). **(A)** OS of patients with NSCLC; **(B)** OS of patients with SCLC; **(C)** PFS of patients with NSCLC; **(D)** PFS of patients with SCLC.

As illustrated in **Figure 4**, the therapeutic efficacy of the ITBP regimen exhibits variability across patients with different pathological subtypes of lung cancer. In patients with NSCLC, the ITBP regimen demonstrates a statistically significant improvement in PFS. Although a favorable trend is observed in OS, this does not reach statistical significance. In contrast, among patients with SCLC, the regimen does not yield statistically significant differences in either OS or PFS, though a potential survival benefit is suggested by the observed trends. These results suggest that the benefits of ITBP may be more pronounced in NSCLC patients, whereas its efficacy in SCLC remains uncertain and requires further validation in larger studies.

#### 3.2.4 Subgroup analysis

**Figure 5:**
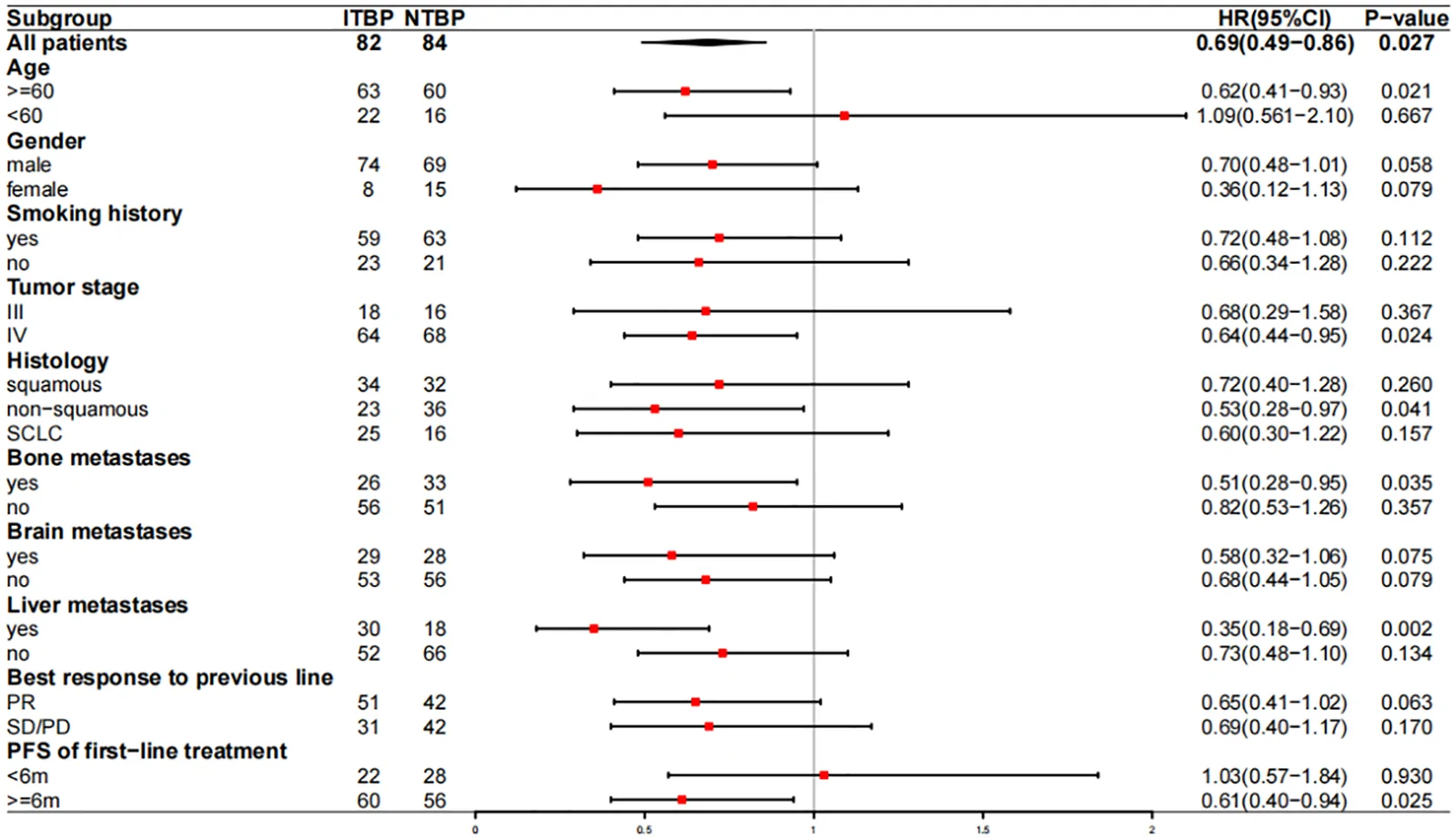
Forest plot for subgroup survival analysis.

To explore the efficacy of ITBP in different patient populations, we conducted a prespecified subgroup analysis of PFS, with the results shown in **Figure 5**. Subgroup analyses were performed to explore the consistency of the survival benefit of ITBP compared with NTBP across clinically relevant patient subgroups (**Figure 5**). Overall, ITBP demonstrated a significant survival advantage over NTBP (HR = 0.69, 95% CI 0.49–0.86, P = 0.027). When stratified by age, patients aged ≥60 years derived significant benefit from ITBP (HR = 0.62, 95% CI 0.41–0.93, P = 0.021), whereas no significant difference was observed in patients <60 years (HR = 1.09, 95% CI 0.56–2.10, P = 0.667). Analysis by tumor stage indicated a significant improvement in stage IV patients (HR = 0.64, 95% CI 0.44–0.95, P = 0.024), but not in stage III. Regarding histology, non-squamous patients exhibited a significant survival benefit (HR = 0.53, 95% CI 0.28–0.97, P = 0.041), whereas squamous cell carcinoma and small-cell lung cancer subgroups did not. Notably, patients with liver metastases (HR = 0.35, 95% CI 0.18–0.69, P = 0.002) or bone metastases (HR = 0.51, 95% CI 0.28–0.95, P = 0.035) experienced the most pronounced benefits. Patients with a longer first-line progression-free survival (≥6 months) also showed significant improvement (HR = 0.61, 95% CI 0.40–0.94, P = 0.025). Other subgroups, including gender, smoking history, brain metastases, and response to prior therapy, showed trends favoring ITBP without reaching statistical significance. Overall, these findings suggest that the survival benefit of ITBP is more pronounced in older patients, those with advanced disease, non-squamous histology, liver or bone metastases, and patients with favorable first-line treatment responses.

#### 3.2.5 PFS risk factors analysis

In univariate Cox regression analysis, patients aged≥60 years exhibited a significantly lower risk of disease progression compared with those aged <60 years (HR = 1.48; 95%CI: 1.01–2.17; P = 0.045). Additionally, first-line PFS≥6 months and treatment with ITBP were both significantly associated with prolonged PFS (HR = 0.56; 95% CI: 0.39–0.80; P = 0.002; and HR = 0.69; 95% CI: 0.49–0.86; P = 0.027, respectively). In contrast, other clinical and demographic variables—including sex, histologic subtype, disease stage, smoking history, and sites of metastasis—were not statistically significant predictors of PFS.

In multivariate Cox regression analysis, first-line PFS ≥6 months (HR = 0.54; 95% CI: 0.37–0.80; P = 0.002) and ITBP treatment (HR = 0.61; 95% CI: 0.42–0.88; P = 0.008) remained independent prognostic factors for improved PFS. By contrast, age was no longer statistically significant (HR = 1.28; 95% CI: 0.84–1.95; P = 0.246), suggesting that the association observed in the univariate analysis was likely confounded by other covariates (**Table 3**).

**Table 3:** Univariate and multivariate analyses of risk factors associated with PFS.

Variables	PFS			
	Univariate analyses		Multivariate analyses	
	HR (95%CI)	*p*	HR (95%CI)	*p*
Age (≥60 vs<60)	1.48(1.01-2.17)	0.045	1.28(0.84-1.95)	0.246
Gender (Female vs Male)	0.83(0.49-1.40)	0.484	0.77(0.43-1.35)	0.356
Histology (SCLC vs NSCLC)	1.13(0.77-1.66)	0.537	1.08(0.69-1.68)	0.752
Stage (IV vs III)	1.24(0.81-1.90)	0.321	1.27(0.79-2.04	0.318
Smoking history (Yes vs No)	1.34(0.91-1.99)	0.141	1.47(0.93-2.32)	0.101
Bone metastasis (Yes vs No)	0.80(0.55-1.17)	0.248	0.75(0.48-1.16)	0.200
Brain metastasis (Yes vs No)	1.01(0.71-1.44)	0.968	1.09(0.72-1.66)	0.672
Liver metastasis (Yes vs No)	1.29(0.90-1.86)	0.185	1.30(0.88-1.92)	0.193
PFS of first-line treatment (PFS≥6m vs<6m)	0.56(0.39-0.80)	0.002	0.54(0.37-0.80)	0.002
Treatment (ITBP vs NTBP)	0.69(0.49-0.86)	0.027	0.61(0.42-0.88)	0.008

## 4 Adverse reactions analysis

IrAEs were generally infrequent and occurred at comparable incidence rates in the ITBP and NTBP groups (**Table 4**). The observed irAEs—including rash, hepatic dysfunction, pneumonitis, thyroid dysfunction, adrenal insufficiency, myocarditis, enteritis, and pancreatitis—were all reported at low frequencies. Notably, hepatic dysfunction occurred significantly more frequently in the NTBP group than in the ITBP group (10.7% versus 2.4%; P = 0.032), whereas no statistically significant intergroup differences were observed for the remaining irAEs. Overall, no novel or unexpected immune-related safety signals emerged. Nevertheless, underreporting—particularly of severe irAEs—remains a potential concern, limiting the study’s ability to comprehensively characterize the risk profile of serious immune-related toxicity.

**Table 4:** Occurrence of irAEs in ITBP and NTBP groups.

Adverse event	Overall incidence of irAEs		
	ITBP (N = 82) (%)	NTBP(N = 84) (%)	*p*
Rash	6 (7.3)	7 (8.3)	0.807
Pneumonia	4 (4.9)	4 (4.8)	0.972
Hepatic dysfunction	2 (2.4)	9 (10.7)	0.032*
Adrenal insufficiency	1 (1.2)	0 (0)	0.310
Enteritis	2 (2.4)	0 (0)	0.150
Myocarditis	1 (1.2)	0 (0)	0.310
Thyroid dysfunction	1 (1.2)	3 (3.6)	0.323
Pancreatitis	0 (0)	1 (1.2)	0.322

* p<0.05 was considered statistically significance.

## 5 Discussion

The results of this retrospective study demonstrate that ITBP offers significant advantages over NTBP in improving OS and PFS, as well as increasing ORR. Further analysis by cancer type indicates a potentially more pronounced therapeutic benefit of ITBP in patients with NSCLC. Importantly, ITBP did not lead to an increased irAEs, suggesting not only enhanced clinical efficacy but also a favorable safety profile, supporting its potential for broader clinical application. Subgroup analyses revealed that patients aged ≥60 years, those at stage IV, non-squamous histology, with liver metastasis, and with a PFS of ≥6 months following first-line therapy derived more substantial benefits from ITBP. These findings suggest that baseline patient characteristics and patterns of PD may influence the effectiveness of ITBP strategies.

In recent years, increasing attention has been paid to the clinical value of ITBP in patients with lung cancer. Wang Yixing et al. (11) reported that, in patients with NSCLC, continuing anti–PD-1 immunotherapy after initial disease progression did not confer additional clinical benefit, and the safety profile was comparable to that of patients who discontinued ICIs. In contrast, Peng Jianfeng et al. (12) demonstrated that among patients continuing immunotherapy beyond progression, those with baseline liver metastases, fewer than three metastatic organs, and no history of smoking experienced more pronounced survival benefits. Although these findings focus on NSCLC, they may also be relevant to extensive-stage small cell lung cancer (ES-SCLC), where approximately 20% of patients develop liver metastases, a subgroup typically associated with poor prognosis, limited chemotherapy responsiveness, and short disease control. In this context, ITBP may confer additional clinical benefit. Li Lingling et al. (13) further suggested that discontinuing immunotherapy immediately after first progression may be premature, particularly for patients who received first-line immunotherapy or showed SD or PD in response to initial therapy. Therefore, decisions regarding cessation of therapy should be considered carefully.

Overall, existing studies indicate that the efficacy of ITBP exhibits considerable heterogeneity and may be closely associated with patients’ baseline characteristics and biological behaviors. This variability can primarily be attributed to several factors. First, between 0.6% and 5.8% of patients with lung cancer undergoing immunotherapy may experience pseudo-progression. Accurate identification of pseudo-progression requires comprehensive evaluation, including clinical manifestations, imaging characteristics, and biomarkers (14). Solely relying on imaging results to determine true progression can lead to premature discontinuation of treatment, depriving patients of potential benefits from continued immunotherapy. Second, tumors may develop resistance to initial immunotherapy, with evolving resistance mechanisms over time. ITBP can address these changing mechanisms, improving treatment efficacy. Additionally, initial immunotherapy may alter the tumor microenvironment, enhancing the ability of subsequent ITBP to penetrate tumor tissue and optimize therapeutic outcomes (15). Third, resistance to immunotherapy may be linked to the loss of tumor antigen expression (16). In such cases, subsequent treatment lines that combine immunotherapy with other modalities (e.g., chemotherapy, radiotherapy, targeted therapy) may improve the immune microenvironment or tumor vasculature, continuing to exert antitumor effects. In summary, ITBP represents a promising option for subsequent treatment in patients with lung cancer. However, clinical decision-making should consider multiple factors, including the patient’s clinical status, prior treatment response, resistance mechanisms, tumor biomarkers, and immune profile. It is also crucial to account for individual characteristics, such as age, comorbidities, and quality of life, while integrating the latest clinical evidence to develop the optimal treatment strategy.

However, Takatoshi Enomoto et al. (17) reported that in patients with advanced NSCLC, continued treatment with nivolumab beyond PD did not yield significant overall clinical benefits. Notably, among patients who did not develop new lesions at the time of first progression, continued nivolumab therapy after progression appeared to provide potential clinical value. These findings suggest that although ITBP may not be universally beneficial, it could remain effective in selected patient subgroups with specific clinical characteristics. One possible explanation is the presence of drug-resistant subclones within the tumor (18). Such subclones may acquire genetic or molecular alterations that enable immune evasion or reduce sensitivity to PD-1/PD-L1 blockade, allowing them to rapidly regain dominance within the tumor microenvironment and thereby limit the efficacy of ITBP. In addition, T-cell exhaustion represents another important mechanism contributing to treatment resistance. Prolonged antigen stimulation within the tumor microenvironment can induce T-cell dysfunction and exhaustion, leading to diminished effector function and impaired tumor recognition and killing (19). Consequently, even with continued immunotherapy in later treatment lines, exhausted T cells may fail to recover sufficient anti-tumor activity, thereby attenuating the expected therapeutic response. Therefore, precise identification of patient populations most likely to benefit from ITBP is essential for optimizing treatment strategies and improving clinical outcomes.

This study has several limitations. First, it is a retrospective analysis, which inherently carries limitations such as dependence on previously recorded medical data for information collection and incomplete follow-up, potentially leading to selection bias and information bias. Second, the relatively short follow-up duration may compromise the accurate assessment of OS and PFS, particularly among long-term survivors. The loss to follow-up or exclusion of certain patients could introduce bias into the survival analyses. Third, although subgroup analyses were performed, imbalances in baseline patient characteristics—such as age, disease stage, presence of liver metastasis, and first-line PFS—were observed, and residual confounding factors may still exist despite these efforts. Fourth, although this study suggests that ITBP may confer significant benefits in specific patient populations, prospective, large-scale randomized controlled trials are warranted to further validate its efficacy and safety.

## 6 Conclusion

This retrospective study demonstrates that ITBP confers significant advantages over NTBP in improving OS, PFS, and ORR, without increasing the incidence of irAEs. The regimen exhibits favorable efficacy and safety profiles, supporting its potential for clinical application. Stratified analyses demonstrated that the survival advantage of ITBP is particularly evident in NSCLC patients aged ≥60 years, with stage IV disease, non-squamous histology, liver metastases, and a first-line PFS of ≥6 months. These findings indicate that baseline patient characteristics and patterns of PD substantially influence the effectiveness of ITBP. Notably, patients with liver metastases also derived meaningful clinical benefit, highlighting that both the presence and absence of specific baseline characteristics can influence the magnitude of ITBP efficacy. These findings underscore the importance of precise patient stratification and individualized treatment planning to optimize outcomes in patients receiving ITBP.

## Data Availability

The original contributions presented in the study are included in the article/supplementary material. Further inquiries can be directed to the corresponding authors.
