# Correction: Clinical benefit and safety of immunotherapy beyond progression in advanced and locally advanced lung cancer: a real-world cohort study

**DOI:** 10.3389/fonc.2026.1897884

**Published:** 2026-07-13

**Authors:** Frontiers Production Office

**Affiliations:** Frontiers Media SA, Lausanne, Switzerland

**Keywords:** advanced or locally advanced lung cancer, disease progression, immune checkpoint inhibitor, immune treatment beyond progression, safety and efficacy

Author “Hua Lan” was erroneously omitted as equal contributing author.

The original version of this article has been updated.

